# Supplementary material for: Liquid-in-liquid printing of 3D and mechanically tunable conductive hydrogels
Source: Nat Commun. 2023 Jul 18;14:4289. doi: 10.1038/s41467-023-40004-7 (PMC10354067; doi:10.1038/s41467-023-40004-7)
Supplement: Supplementary file 1 — Supplementary Information [file 41467_2023_40004_MOESM1_ESM.pdf]

Supplementary Information for

Liquid–in–Liquid Printing of 3D and Mechanically Tunable Conductive Hydrogels

Xinjian Xie,[1] *Zhonggang Xu,* [1] *Xin Yu,*[2] *Dr. Hong Jiang,* [2] *Dr. Hongjiao Li*,*[3]
and Dr. Wenqian Feng[1,4]

[1] College of Polymer Science and Engineering, Sichuan University, 610065 Chengdu, China

[2] Department of Pancreatic Surgery, Department of Biotherapy, West China Hospital, Sichuan University, 610065 Chengdu, China

[3] College of Chemical Engineering, Sichuan University, 610065 Chengdu, China

[4] State Key Laboratory of Polymer Materials Engineering, Sichuan University, 610065 Chengdu, China

* Correspondence to: hongjiao.li@scu.edu.cn; feng.wenqian@scu.edu.cn

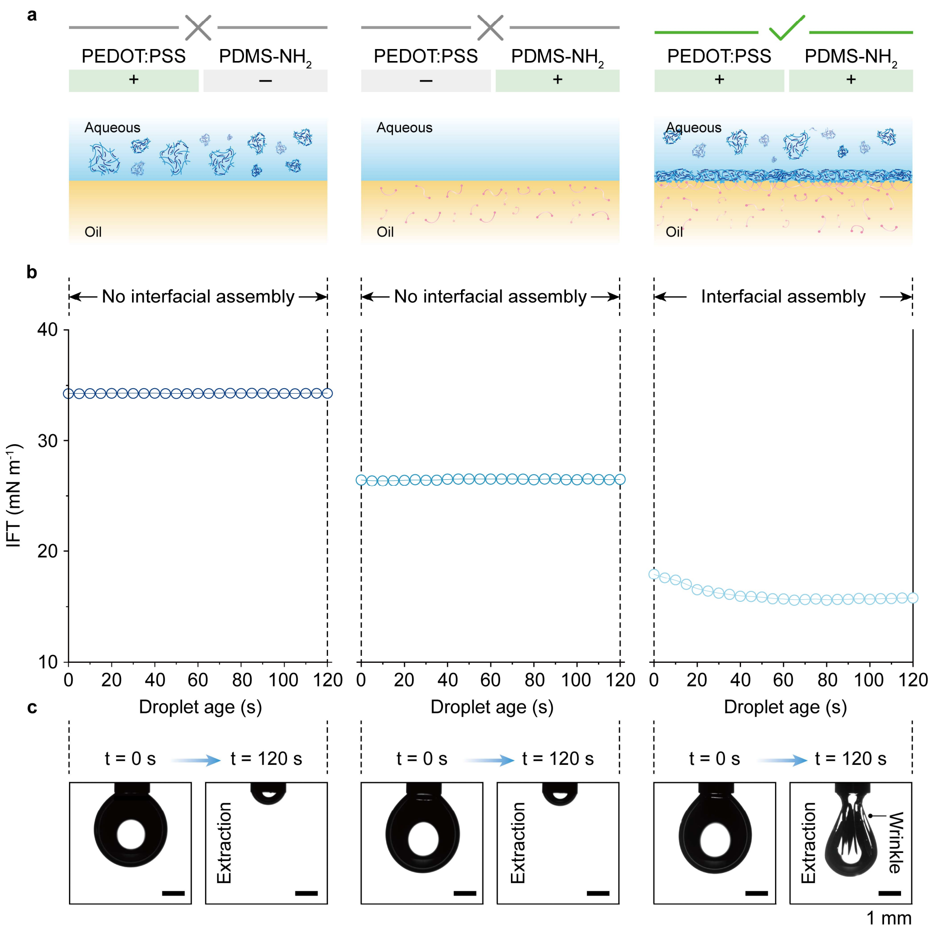

Supplementary Fig. 1 | PEDOT:PSS-surfactants (PPSs) assembly behavior at the liquid-liquid interface. a,b The presence of PEDOT:PSS in the aqueous phase alone does not exhibit strong interfacial activity, while the amine end-capped silicone oil ($PDMS\text{-}NH_2$) in the oil phase possesses a low degree of interfacial activity (as indicated by its IFT value at the steady state). When both components are present in each phase simultaneously, the interfacial tension decreases dramatically and reaches a low value. It is reasonable to conclude that the amine-capped silicone oil initially assembles at the interface in the form of $PDMS\text{-}NH_3^+$, and the negatively charged PEDOT:PSS particles then quickly diffuse to the interface and electrostatically interact with the amine end-groups on the silicone oil to form the PPSs interfacial assembly. **c** The droplet morphology and its buckling behavior when the PEDOT:PSS dispersion is withdrawn. Well-defined wrinkles are only observed at the interfacial area with PPSs assembly.

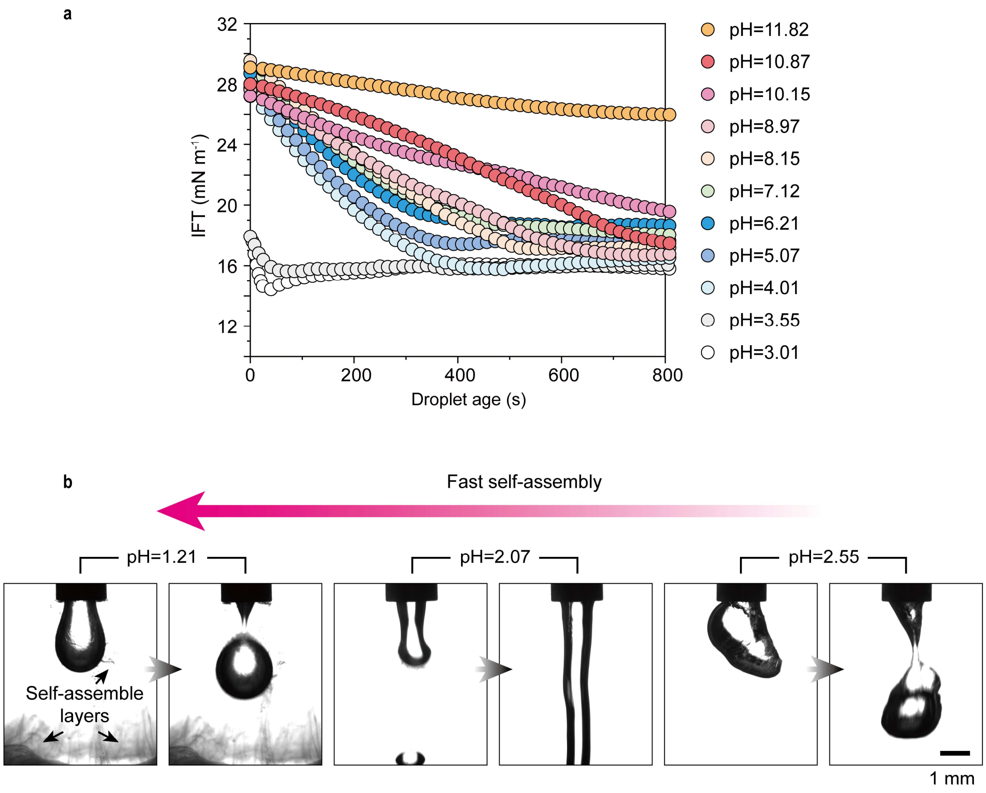

Supplementary Fig. 2 | pH-dependent PEDOT:PSS-PDMS surfactant interfacial assembly (PPSs). a Temporal evolution of the interfacial tension (IFT) of aqueous PEDOT:PSS dispersions (0.5 mg mL^{-1}, pH range of 3.01–11.82) introduced into solutions of PDMS-NH_2 in toluene (10 vol%). **b**. Droplet morphologies of PEDOT:PSS dispersion (0.5 mg mL^{-1}, pH 1.21–2.55) introduced into solutions of PDMS-NH_2 in toluene (10 vol%). At pH lower than 2.55, the initial adsorption of PEDOT:PSS particles to the interface is too rapid to form regular droplet shapes for IFT measurements. Adjusting the pH further to 1.21, the PEDOT:PSS droplet became opaque and parts of the assembly containing PEDOT:PSS were removing from the aqueous phase into oil. This may arise from the formation of micelles (water in oil (W/O)) at the liquid phase, due to the very low interfacial tension and osmotic pressure at low pH.[1]

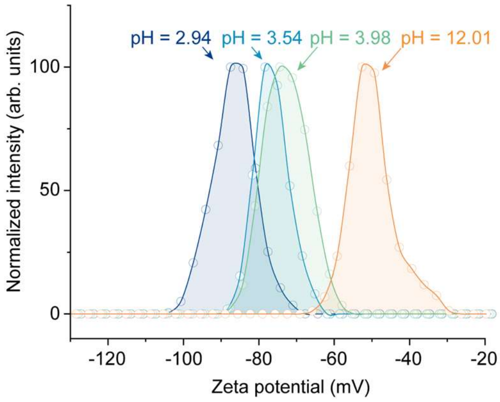

Supplementary Fig. 3 | Variation of zeta-potential of PEDOT:PSS aqueous solution with pH.

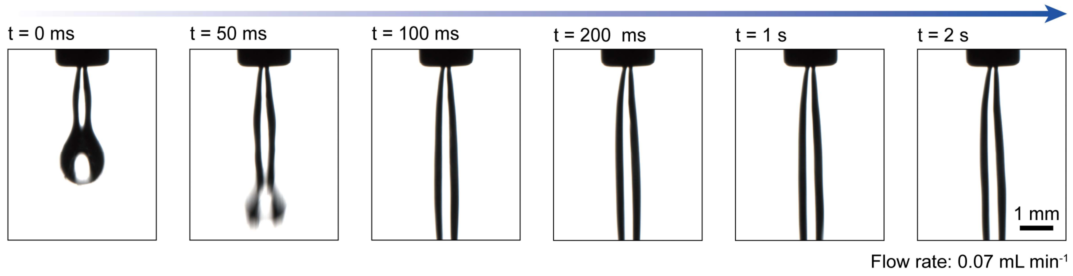

Supplementary Fig. 4 | A continuous jet without breakup when introducing a PEDOT:PSS dispersion (0.5 mg mL^{-1}, pH 2.07) into a solution of PDMS-NH$_2$ in toluene at a flow rate of 0.07 mL min^{-1}.

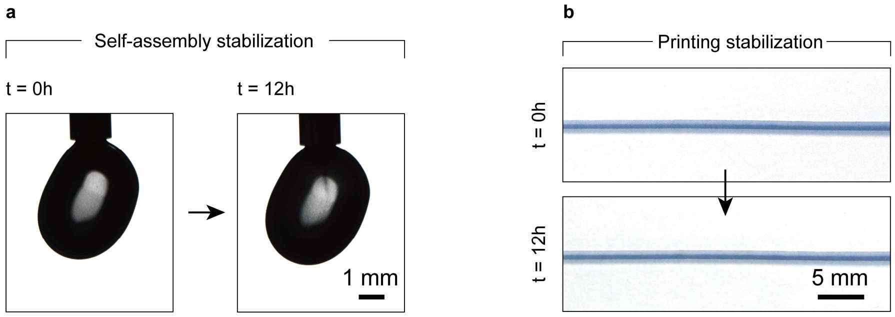

Supplementary Fig. 5 | The ability of instantaneous interfacial PPSs to maintain the original shape of droplets or liquid threads. a) A pendent PEDOT:PSS droplet (1 mg mL^{-1} aqueous dispersion) in toluene solution of PDMS-NH$_2$ surfactants (10 vol%) reaching its steady state within milliseconds and maintaining this state for at least 12 hours. b) A printed PEDOT:PSS thread (0.5 mg mL^{-1} aqueous dispersion) in oil phase (10 vol% PDMS-NH$_2$ in silicone oil, η_e ~ 30000 mPa·s) maintaining its shape for 12 hours.

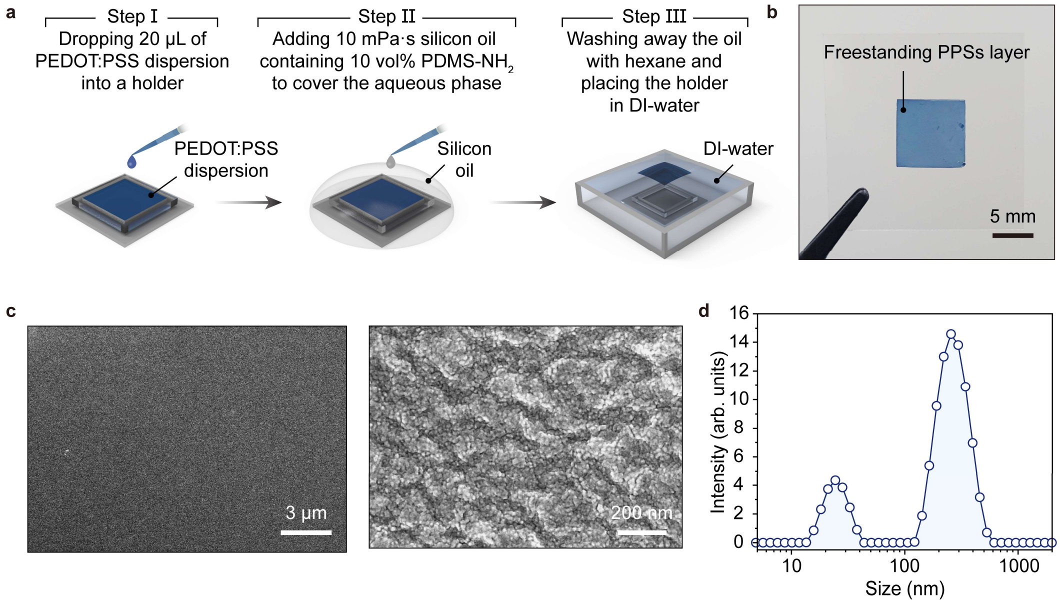

Supplementary Fig. 6 | Visualizing the PPSs interfacial assembly. **a** Formation of a planar layer of PPSs at the liquid-liquid interface in a mold. When the aqueous and oil phases are brought into contact, interfacial assembly occurs, resulting in the formation of a flat and uniform PPSs film at the interface. The assembled layer remains stable in water due to the cross-linked structure, allowing it to be easily transferred to a glass substrate. **b** PPSs assembly layer collected on glass substrate. **c** SEM images showing the densely packed PEDOT:PSS nanoparticles at the assembly layer. Similar results were observed in two independent samples. **d** Size distribution of PEDOT:PSS particles by intensity, measured at ~pH 3.5 using dynamic light scattering (DLS).

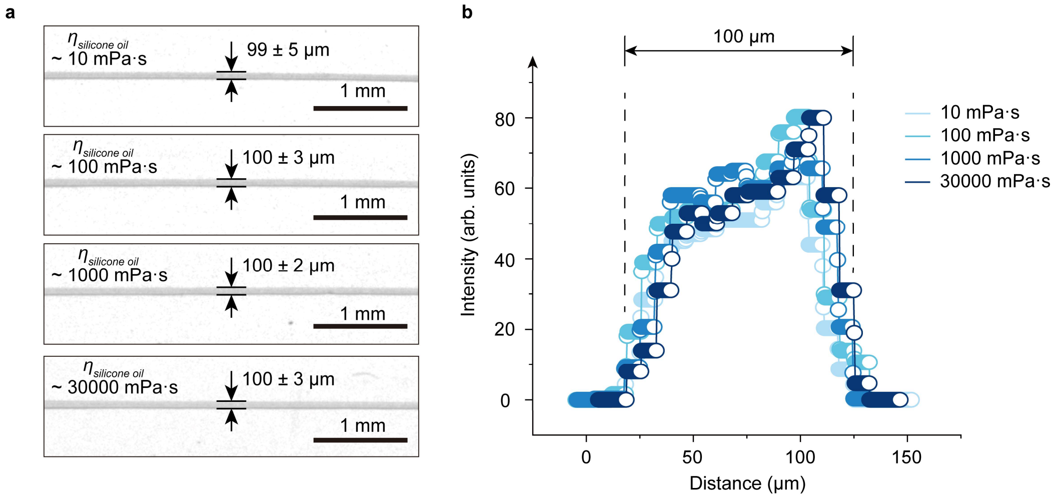

Supplementary Fig. 7 | Diameter control of printing pure PEDOT:PSS threads in oil phase with various viscosities. a Optical images of the PEDOT:PSS aqueous dispersion (10 mg mL^{-1}) injected in various silicone oil containing 10 vol% PDMS-NH_2 surfactants. **b** Diameter distribution of the printed PEDOT:PSS aqueous dispersion (10 mg mL^{-1}) in different silicone oil with various viscosity. The diameter of the printed PEDOT:PSS threads in cylindrical shape is only related to print head speeds (ν) and ink volumetric flow rate (Q) but not to the oil viscosity. The threads thickness can also be calculated by the following formula:

$$d = 2\sqrt{\frac{Q}{\pi\nu}}$$

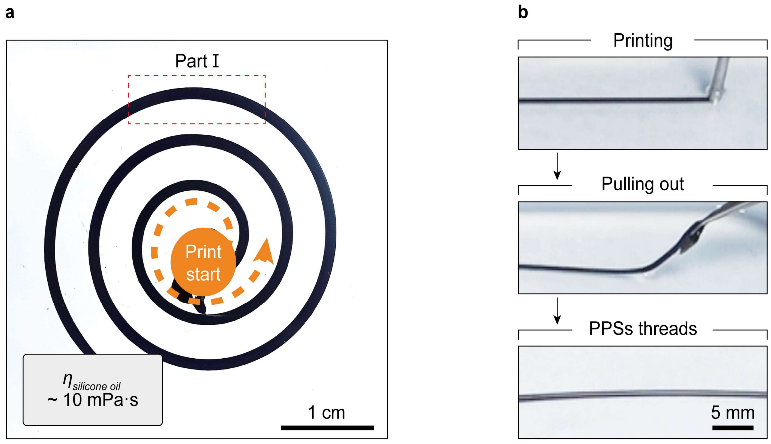

Supplementary Fig. 8 | a The quick PPSs assembly at the liquid-liquid interface enables printing of liquid threads in low-viscous oil ($\eta_e \sim$ 10 mPa·s). **b** The PPSs interfacial assembly showing superior mechanical robustness. The PPSs assembly exhibits strong mechanical strength and deformation resistance, which is achieved through the PDMS chains (in this study PDMS surfactants are capped on both ends with primary amines, i.e., NH_3^+-PDMS-NH_3^+at the liquid interface) that bridge adjacent

PEDOT:PSS nanoparticles to form an effectively cross-linked, densely packed, and solid-like interfacial assembly layer.[2]

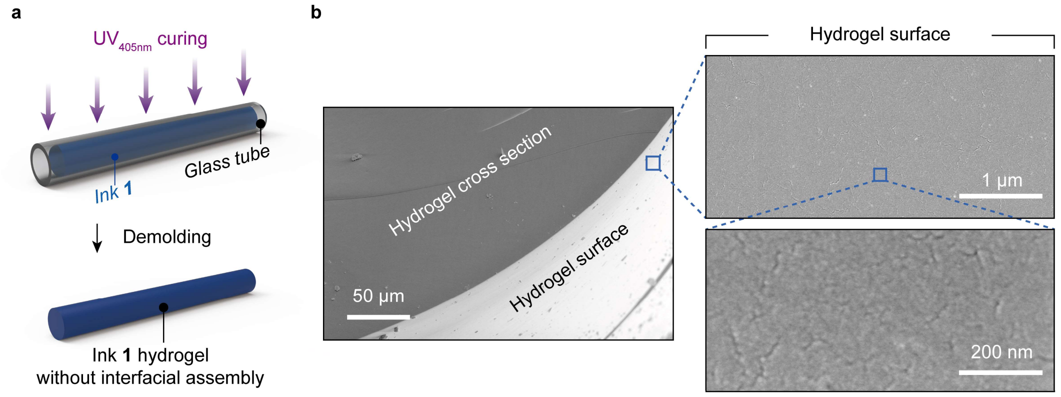

Supplementary Fig. 9 | Fabrication of PEDOT@PEG hydrogel without PPSs interfacial assembly. a Schematic representation of UV-curing of a PEDOT@PEG hydrogel (ink 1) in a glass tube. **b** Cross-sectional SEM images of the resulting PEDOT@PEG gel (ink 1) after freeze-drying. Similar results were observed in two independent samples. The absence of PPSs interfacial assembly in the fabrication process results in a lower PEDOT:PSS density on the gel's surface, in contrast to the PPSs assembly layer shown in Supplementary Fig. 6c. It is reasonable to attribute the skin-core structure shown in Figure 3c to differences in acrylate monomer concentration between the liquid filament's shell and core, which result from the interfacial self-assembly of PEDOT:PSS-surfactants (PPSs) during the printing process.

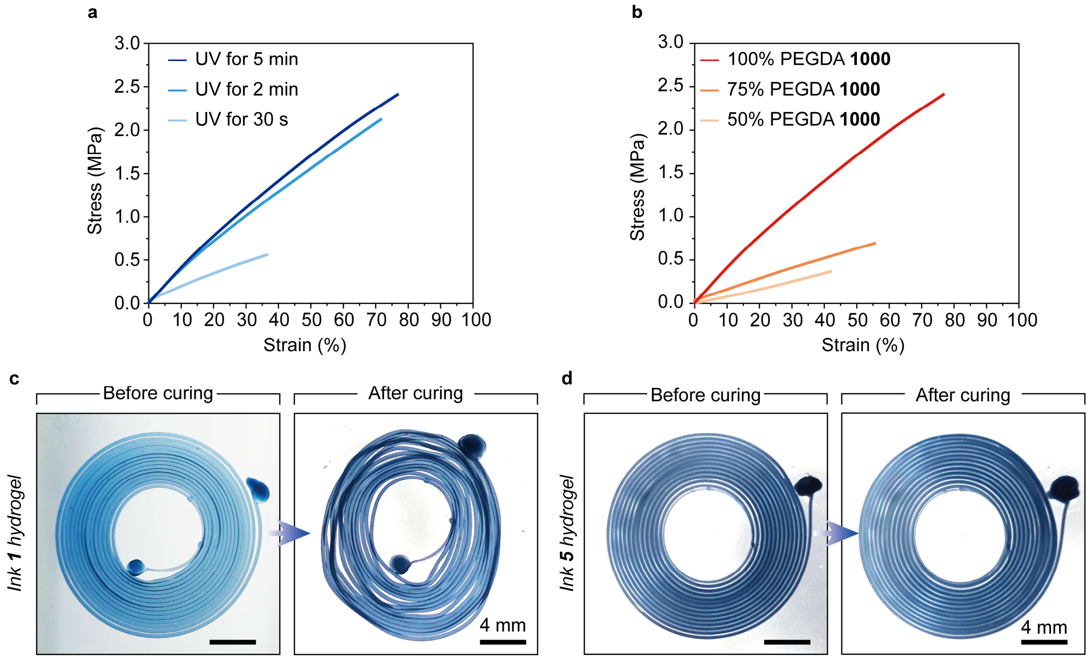

Supplementary Fig. 10 | Tuning the elastic moduli of the printed hydrogels (ink 1) by varying the UV curing time **a** and monomer concentration **b**. The shrinkage of

hydrogel-forming precursors after UV curing is of different degrees to different formulations (**c** and **d**).

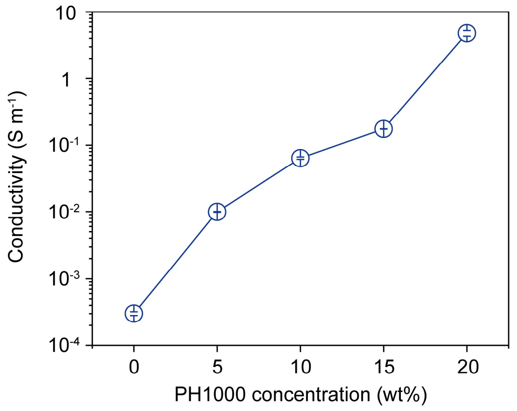

Supplementary Fig. 11 | Electrical conductivity of the printed hydrogels (385 mg mL^{-1} PEG 1000 monomer, 5 min UV irradiation) with different PEDOT:PSS concentration. Although the conductivity of such hydrogels increases as a function of PEDOT:PSS concentration in the hydrogel-forming precursor, the increase in PEDOT:PSS concentration results in a sharp rise in apparent viscosity of the polymer inks. The viscosity of the ink with 20 wt% PEDOT:PSS is over 23000 mPa·s. Values represent the mean, and the error bars represent the SD of the measured values (n = 5).

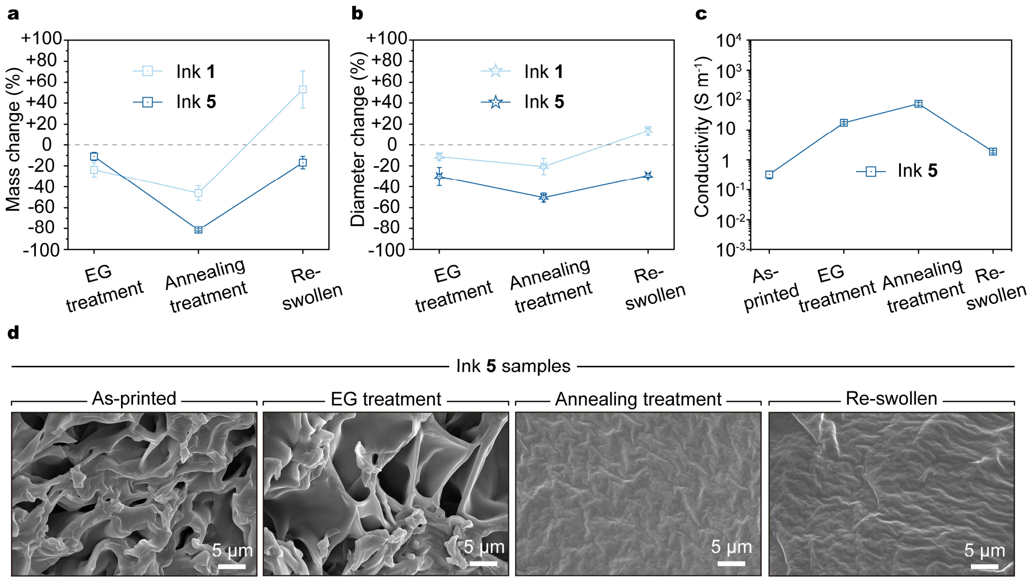

Supplementary Fig. 12 | Mass **a**, dimeter **b**, electrical conductivity **c** and polymer morphology **d** changes of the printed hydrogels in post-treatment process. The similar results were observed across two independent samples within each group in **d**. The water content values in the hydrogels can be calculated by dividing the difference between the dried (W_{dry}) and swollen ($W_{swollen}$) weights by the swollen state weight, i.e., water content = ($W_{swollen}$ - W_{dry}) / $W_{swollen}$. Such reconfiguration of the polymer

morphology in post-treatment changed the swelling ability of polymers as well. The as-synthesized gel from ink 5 swelled to approximately 2.6 times its original weight, whereas the post-treated ink 5* gel underwent a reswelling process in water with only 85% reswollen weight. Values in **a**, **b**, and **c** represent the mean, and the error bars represent the SD of the measured values (n = 3).

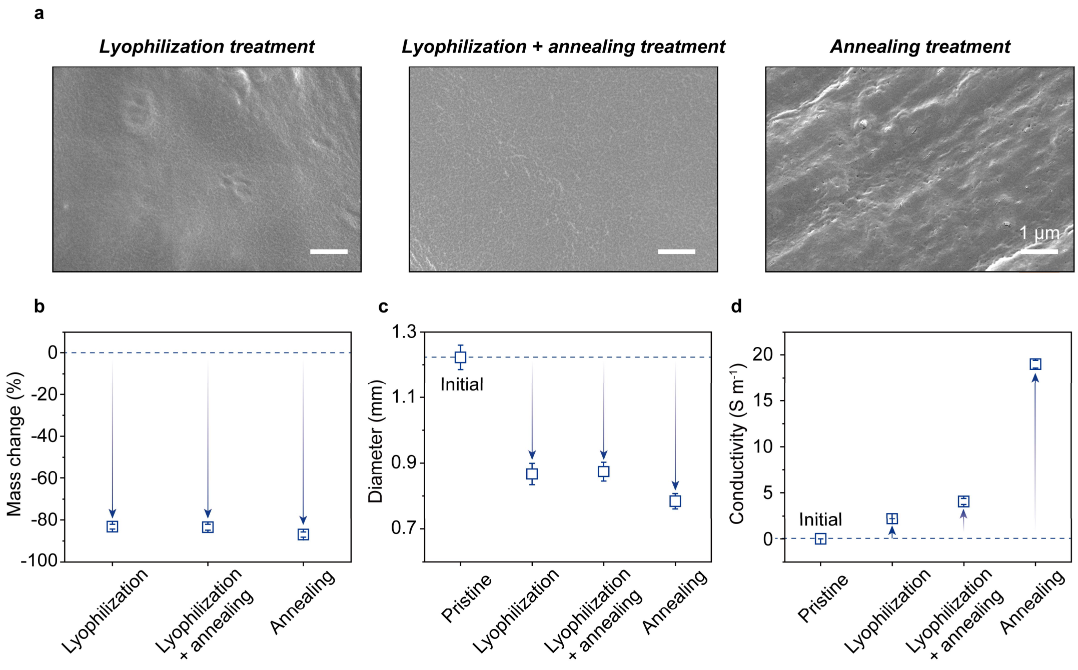

Supplementary Fig. 13 | Comparison of different drying treatments on printed hydrogels (ink 5): lyophilization, lyophilization followed by annealing, and direct dry annealing. a Polymer morphology changes of the printed hydrogels (ink 5) after drying treatments. To eliminate other influencing factors, the printed hydrogel samples were directly dried without removing the uncured monomers. While some degree of pore collapse is observed for all three drying treatments, the directly dry-annealed samples show a higher degree of network stacking and aggregation, which is also illustrated in the diameter variation of the samples. The similar results were observed across two independent samples within each group in **a**. **b** Changes in mass, diameter, and electrical conductivity of the printed hydrogels during different drying treatments. While the amount of water loss is almost the same for all treatments, it's reasonable to conclude that the water-loss-induced collapse of the conducting PEDOT@PEG network into aggregates in the dry annealing process is the main contributor to the enhanced conductivity of the printed gels. Values in **b**, **c**, and **d** represent the mean, and the error bars represent the SD of the measured values (n = 3).

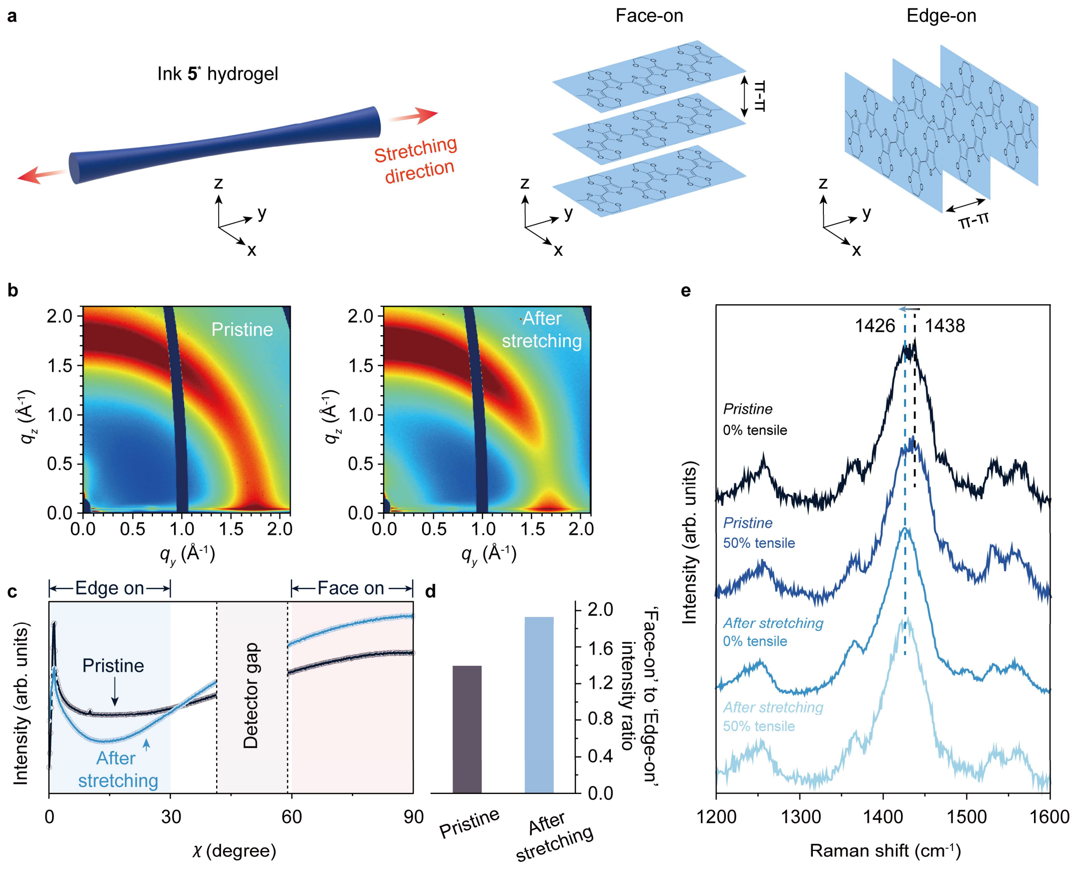

Supplementary Fig. 14 | Conformational changes of the PEDOT@PEG gels after cyclic stretching. a Schematic illustration of molecule orientations in edge-on and face-on configurations, with respect to the direction of polymer gel stretching. **b** 2D GIWAXS measurements of printed PEDOT@PEG gels (ink 5*) before and after reversible stretching of the gel between 0 and 50% strain. The printed PEDOT@PEG gels exhibit the similar characteristic peak to the pristine PEDOT:PSS material. **c** Ascription of face-on and edge-on regions by the χ angle: edge-on region ($0° < \chi < 30°$), face-on region ($60° < \chi < 90°$). **d** Intensity ratio change of face-on to edge-on orientations. To investigate the molecular orientation, sector integrals from horizontal (0°-30°) to vertical (60°-90°) direction were calculated for the printed PEDOT@PEG gel before and after cyclic stretching to 50% strain. As scattering from the π–π stacking of the PEDOT:PSS molecules in horizontal and vertical directions characterizes the edge-on and face-on orientations of the molecules relative to the stretching direction, respectively, the PEDOT:PSS crystallites changed from a disordered arrangement to a preferred face-on molecular orientation during stretching, as evidenced by the noticeable increment of the face-on to edge-on integration ratio. **e** Raman spectra illustrating the $C_\alpha = C_\beta$ vibration peak shift for the PEDOT@PEG gels (ink 5*) after reversible stretching treatment. This shift indicates a higher proportion of the benzoid moieties in PEDOT have been converted to the quinoid structure via oxidative charge transfer doping, and such conversion leads to a more planar backbone, contributing to more efficient charge delocalization and higher packing order.[3, 4]

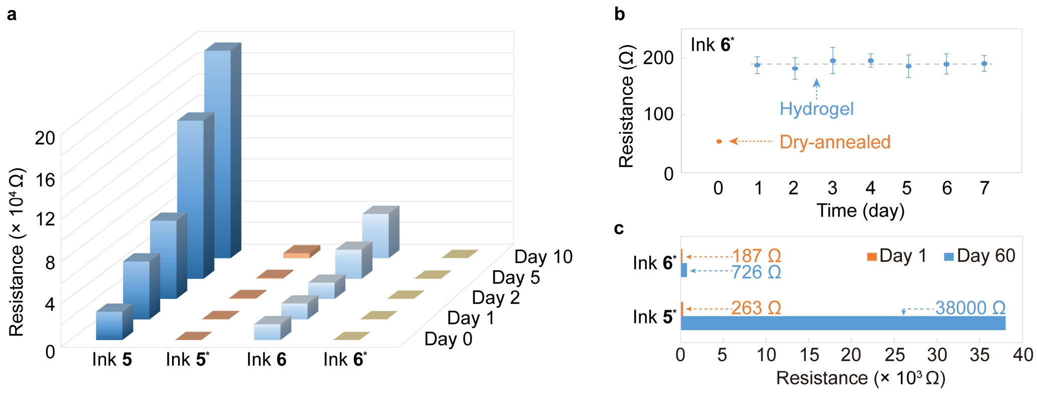

Supplementary Fig. 15 | Electrical stability of the printed gels in liquids. a Resistance change of the gels (ink 5, ink 5*, ink 6, ink 6*) when soaked in DI water for certain days. **b** Gel ink 6* showing good electrical stability over 7 days. **c** The long-term electrical stability of gel ink 5* and ink 6*. Both post-treatment (comparation between ink 5 and ink 5*, or between ink 6 and ink 6*, shown in **a**) and ionic liquid-induced gelation (comparation between ink 5* and ink 6*, shown in **c**) are capable of improving the electrical conductivity and stability of the resultant gels considerably. 1-cm-long cylindrical samples were used for measuring resistance under various conditions. Values in **b** represent the mean, and the error bars represent the SD of the measured values (n = 3).

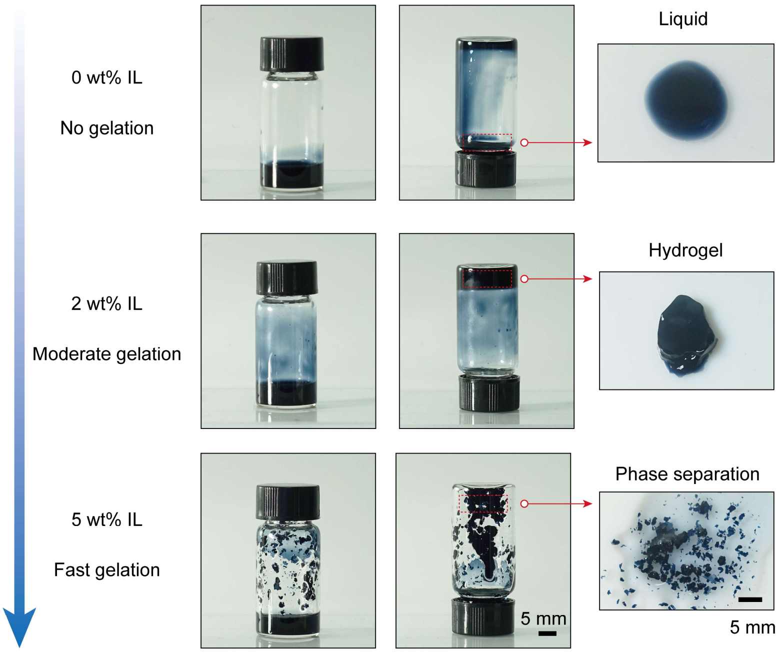

Supplementary Fig. 16 | Gelation of liquid PEDOT ink 5 when mixed with varying amounts of ionic liquid (IL) HOOCMIMNTF$_2$**.** The ink 5 containing 2 wt% IL undergoes gelation at a moderate rate, whereas the one consisting of 5 wt% IL has an irreversible phase separation after 10 min. The similar results were observed across two independent samples within each group.

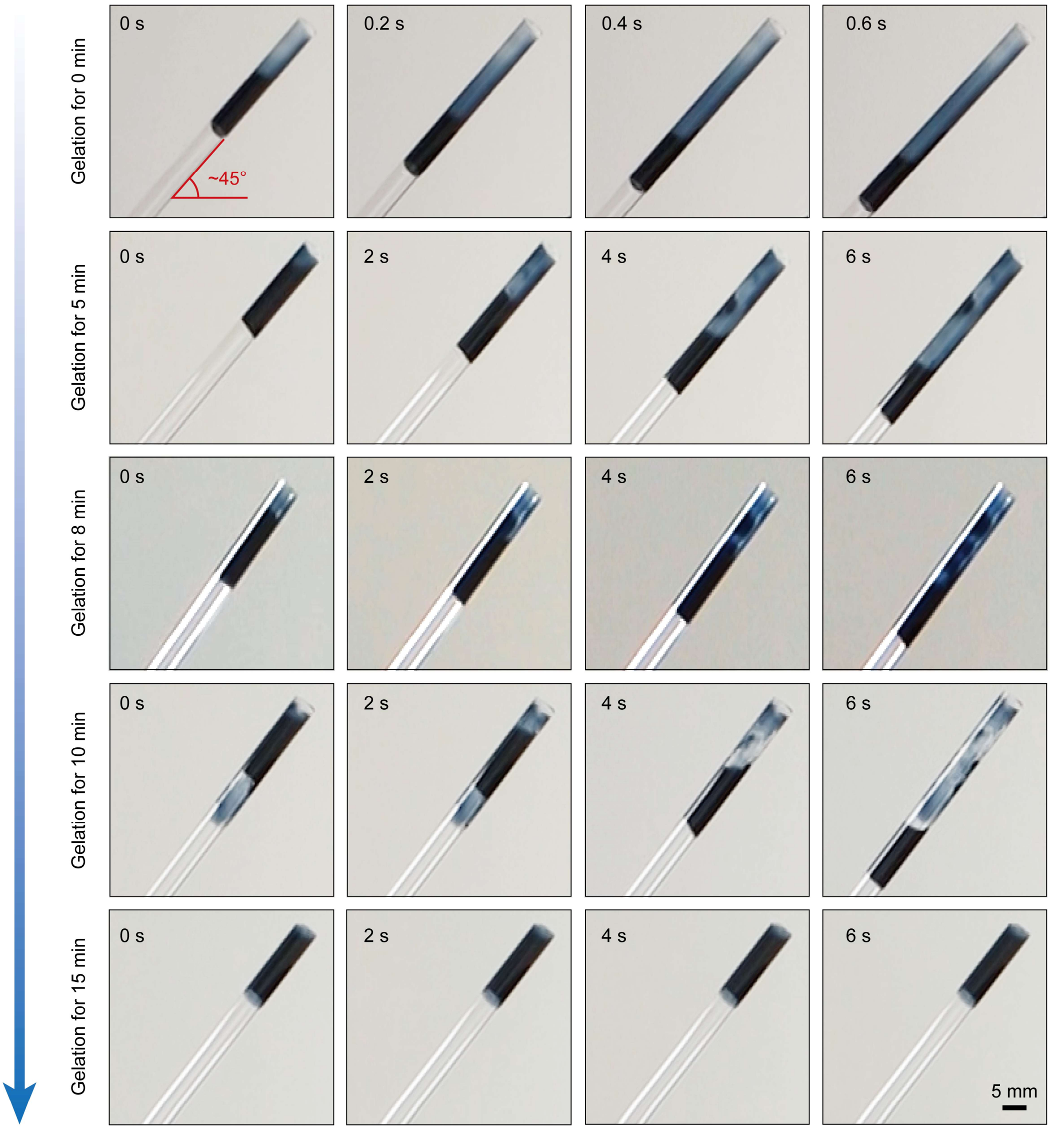

Supplementary Fig. 17 | Flowability of the PEDOT ink when mixed with 2 wt% ionic liquid HOOCMIMNTF$_2$ (ink 6). 200 µL fresh ink 6 was injected into a glass tube and left to sit for a specified time period. The tube was then tilted at a 45-degree angle to observe the ink's flow behavior. The similar results were observed across two independent samples within each group.

Given that the inside diameter of the tube is 1.5 mm, the capillary force is negligible compared to the gravity of the injected ink liquid. When the physical gelation time is less than 10 minutes, the ink in the tube remains in a liquid state and flows under the action of gravity, with some liquid remaining on the tube wall. However, when the gelation time is prolonged to 15 minutes, the ink appears as a bulk and loses its fluidity in the glass tube. Based on this observation, we conclude that the ink mixture can maintain its flowability for at least the first 8 minutes after adding the ionic liquid.

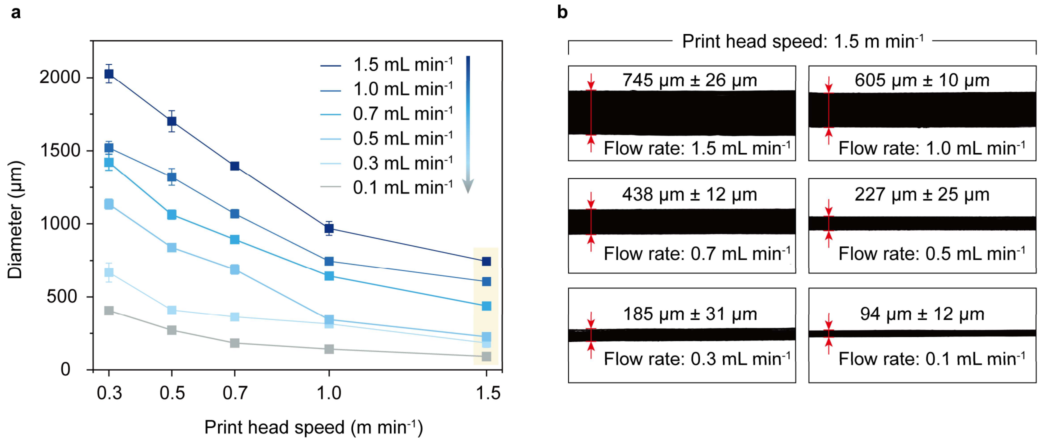

Supplementary Fig. 18 | Diameter control of the printed hydrogels. a Readily modulation of the diameter of the cured hydrogels by adjusting the print head speeds and ink flow rates. **b** Printed hydrogel filaments of varying diameters. Values in **a** represent the mean, and the error bars represent the SD of the measured values (n = 3).

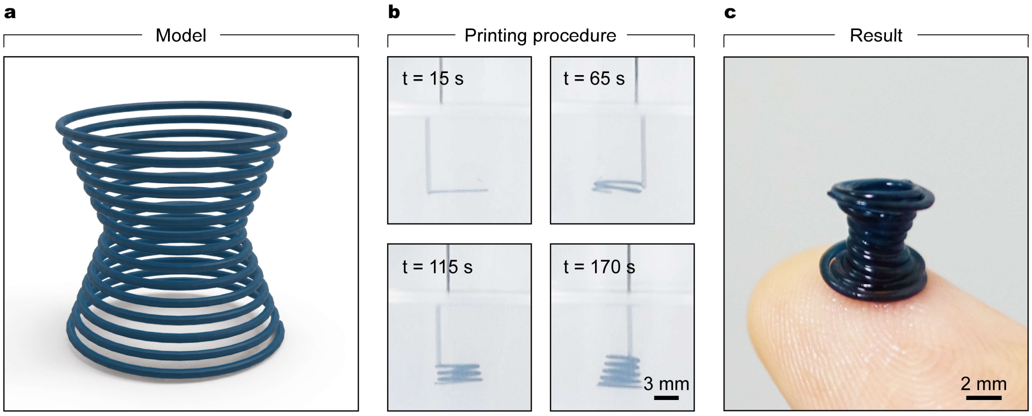

Supplementary Fig. 19 | Printing of hydrogels with 3D microstructure. a Schematic of the 3D printing model. **b** Time lapse of the printing process. **c** A cured conductive hydrogel with 3D hourglass shape.

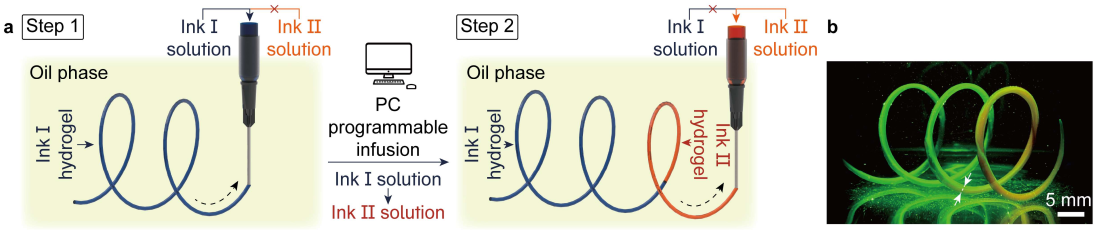

Supplementary Fig. 20 | a Integration of multiple materials into hydrogels by programming the ink feeds. **b** Optical image of a helix 3D hydrogel consisting of two components.

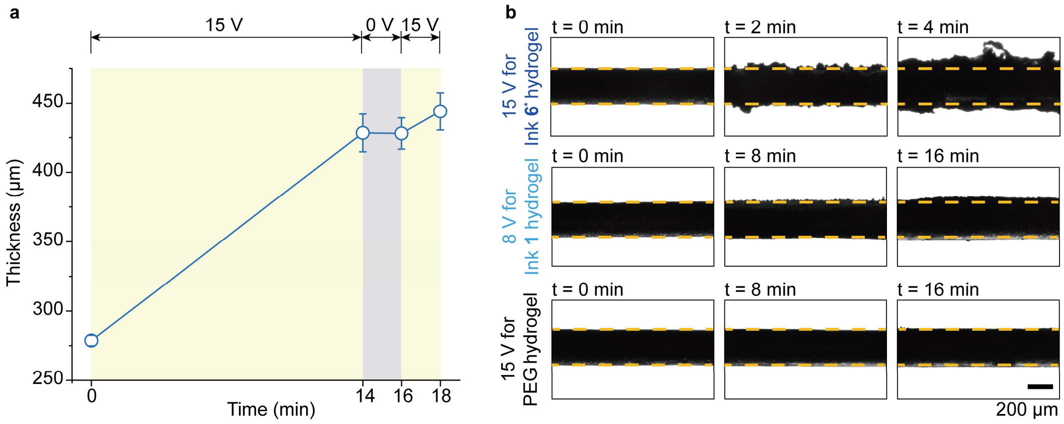

Supplementary Fig. 21 | Copper electroplating in the PEDOT-based tubular hydrogels. a Plot of the surface thickness variation of the Pt wire in the PEDOT-based electrochemical microfluidic device. The reduction of Cu(II) ions in electrolyte flow results in the deposition of Cu(0) on the Pt cathode surface with the supply of power, whereas there was no copper deposition onto the Pt during the power outage (grey regime). **b** Evolution of the microstructure of the Pt cathode during electrolysis under different applied voltages. Values in **a** represent the mean, and the error bars represent the SD of the measured values (n = 5). All Pt wires undergo identical electrolysis processes.

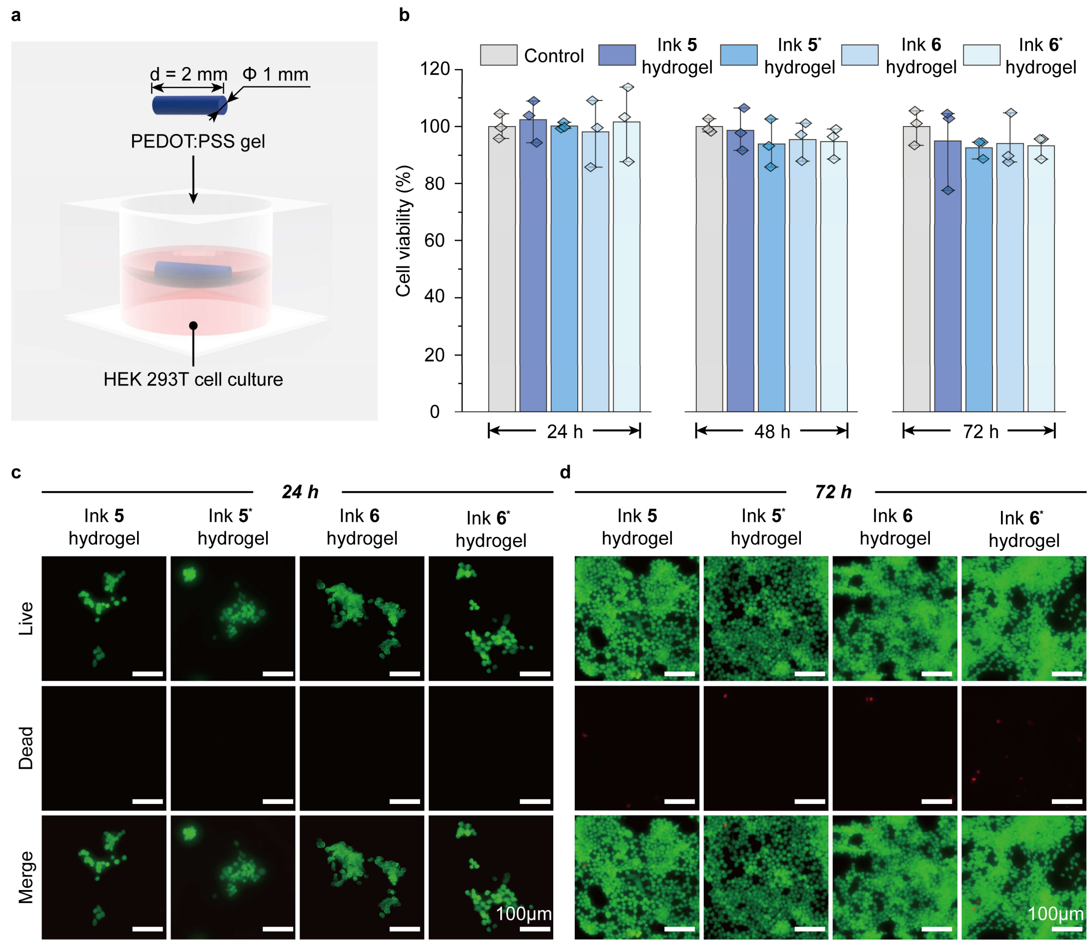

Supplementary Fig. 22 | *In vitro* cytocompatibility tests of immortalized human embryonic kidney cells (HEK 293T cells) with printed PEDOT:PSS hydrogels. a Schematic setup of the cell culture in 96-well plates with various printed PEDOT:PSS hydrogels. **b** Cell viability measured after 24, 48, and 72 hours of culture using Cell Counting Kit 8 (CCK8) assay. Control cells were cultured in a medium without hydrogels. **c** Live/Dead staining of HEK 293T cells at 24 and 72 hours for cell viability and proliferation evaluation. At the end of the 72-hours culture period, the cell viability percentage was almost equal (~95%) across all hydrogel samples. The increasement in cell proliferation during cell culture for all the samples also demonstrates the well cytocompatibility of the printed PEDOT-based hydrogels. Values in **b** represent the mean, and the error bars represent the SD of the measured values (n = 3). The similar results were observed across two independent samples within each group in **c** and **d**.

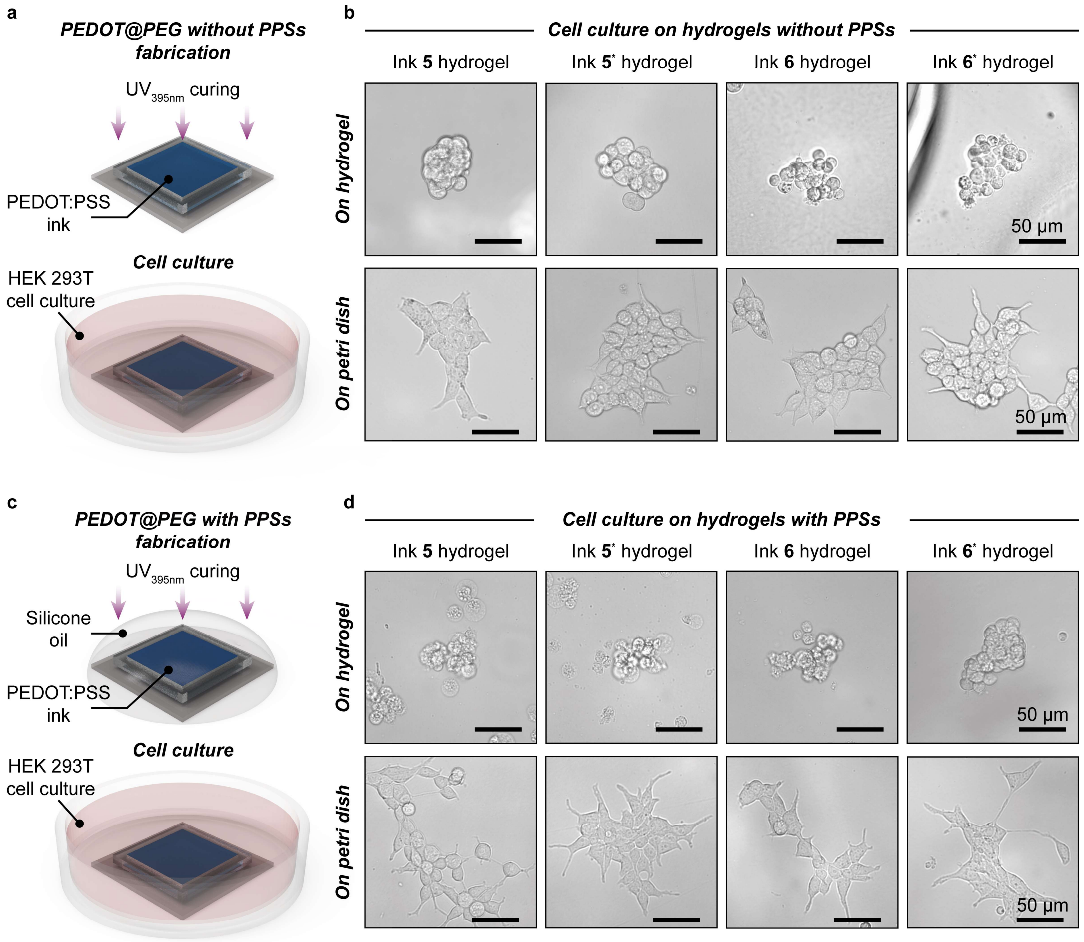

Supplementary Fig. 23 | Evaluation of the anti-adhesive effect of PEDOT@PEG hydrogels (ink 5, ink 5*, ink 6 and ink 6* formulas) with and without PPSs interfacial assembly on HEK 293T cells. a Schematic showing the fabrication of PEDOT@PEG hydrogels without PPSs interfacial assembly in a mold and subsequent cell seeding on hydrogel surfaces. **b** Morphology of HEK 293T cells in direct contact with PEDOT@PEG hydrogels without PPSs interfacial assembly after 72 hours of culture time. **c** Schematic showing the fabrication of PEDOT@PEG hydrogels with PPSs interfacial assembly in a mold and subsequent cell seeding on hydrogel surfaces. **d** Morphology of HEK 293T cells in direct contact with PEDOT@PEG hydrogels with PPSs interfacial assembly after 72 hours of culture time. At the end of the 72-hours culture, the difference in cell spreading between the hydrogel and the surrounding petri dish demonstrates that both PEDOT@PEG hydrogels with and without PPSs interfacial assembly possess anti-adhesive effects on cells. The similar results were observed across two independent samples within each group in **b** and **d**.

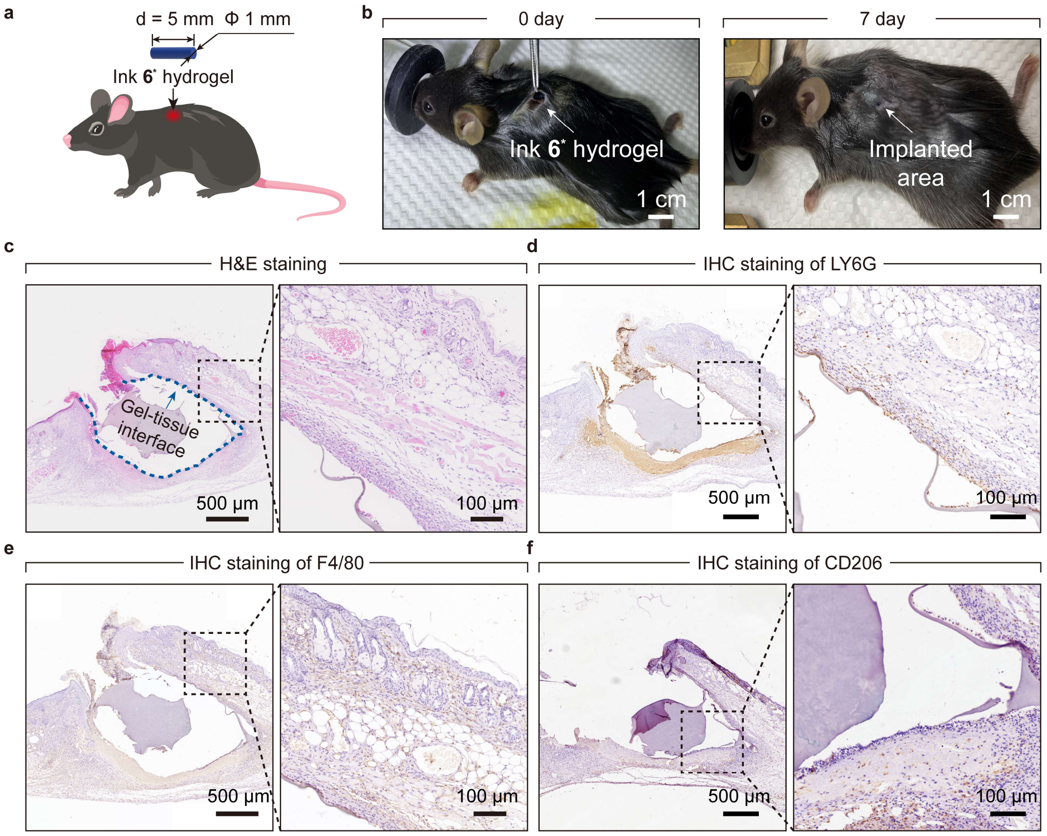

Supplementary Fig. 24 | *In vivo* assessment of hydrogel biocompatibility in immunocompetent mice. a,b Schematic diagram and photograph depicting the subcutaneous implantation of printed hydrogels (ink 6*), which is the formula that contains the most components. **c** Pathological study through H&E staining on the implantation site 7 days after surgery. **d-f** Representative immunohistochemistry (IHC) staining of markers for neutrophils (LY6G), pan-macrophages (F4/80), and M2 macrophages (CD206), respectively. The blue line indicates the hydrogel-tissue interface. The ratio of M2 macrophages to pan-macrophages (> 66%) around the hybrid PEDOT@PEG hydrogel suggests that the macrophages mainly display alternative activation and, therefore, exhibit an anti-inflammatory response, supporting tissue repair. The printed PEDOT@PEG hydrogels exhibit high biocompatibility in immunocompetent mice at this time point, as the mice remained alive and healthy without any abnormalities for two months after subcutaneous implantation. Longer-term monitoring of the foreign body reaction in the mice is still ongoing. The similar results were observed across four independent samples within each group in **c**, **d, e,** and **f**.

Supplementary Notes: We have complied with all relevant ethical regulations about the research animal. The board and institution that approved the study protocol in the manuscript: Department of Biotherapy, West China Hospital, Sichuan University.

PEDOT:PSS concentration (mg mL^{-1})	pH value	Zeta potential (mV)
0.5	2.94	-85.0 ± 4.52
0.5	3.54	-77.5 ± 1.36
0.5	3.98	-76.5 ± 7.28
0.5	12.01	-47.1 ± 2.48

Supplementary Table 1 | Zeta potential variability in PEDOT:PSS aqueous dispersions with different pH values.

Supplementary References

1. Bae J, Russell TP, Hayward RC. Osmotically Driven Formation of Double Emulsions Stabilized by Amphiphilic Block Copolymers. *Angew Chem Int Ed* **53**, 8240-8245 (2014).

2. Cui M, Emrick T, Russell Thomas P. Stabilizing Liquid Drops in Nonequilibrium Shapes by the Interfacial Jamming of Nanoparticles. *Science* **342**, 460-463 (2013).

3. Garreau S, Louarn G, Buisson JP, Froyer G, Lefrant S. In Situ Spectroelectrochemical Raman Studies of Poly(3,4-ethylenedioxythiophene) (PEDT). *Macromolecules* **32**, 6807-6812 (1999).

4. Wang Y*, et al.* A highly stretchable, transparent, and conductive polymer. *Science Advances* **3**, e1602076.
