# Supplementary material for: Liquid-in-liquid printing of 3D and mechanically tunable conductive hydrogels
Source: Nat Commun. 2023 Jul 18;14:4289. doi: 10.1038/s41467-023-40004-7 (PMC10354067; doi:10.1038/s41467-023-40004-7)
Supplement: Supplementary file 3 — Description of Additional Supplementary Files [file 41467_2023_40004_MOESM3_ESM.docx]

**List and description of supplementary movies**

**Supplementary Movie 1:** Liquid–in–liquid printing of a 1.1 m-long conductive gel in a 1.8 cm-diameter circle of printing area.

**Supplementary Movie 2:** Liquid–in–liquid 3D printing of PEDOT-based liquid inks with overhung structures.

**Supplementary Movie 3:** Wireless power transmission on the PEDOT-based NFC chip.
