# Supplementary material for: Liquid-in-liquid printing of 3D and mechanically tunable conductive hydrogels
Source: Nat Commun. 2023 Jul 18;14:4289. doi: 10.1038/s41467-023-40004-7 (PMC10354067; doi:10.1038/s41467-023-40004-7)
Supplement: Supplementary file 7 — Reporting Summary [file 41467_2023_40004_MOESM7_ESM.pdf]

nature portfolio

Corresponding author(s): Hongjiao Li, Wenqian Feng

Last updated by author(s): Jun 27, 2023

Reporting Summary

Nature Portfolio wishes to improve the reproducibility of the work that we publish. This form provides structure for consistency and transparency in reporting. For further information on Nature Portfolio policies, see our Editorial Policies and the Editorial Policy Checklist.

Statistics

For all statistical analyses, confirm that the following items are present in the figure legend, table legend, main text, or Methods section.

n/a	Confirmed	
☐	☒	The exact sample size (n) for each experimental group/condition, given as a discrete number and unit of measurement
☐	☒	A statement on whether measurements were taken from distinct samples or whether the same sample was measured repeatedly
☒	☐	The statistical test(s) used AND whether they are one- or two-sided<br>*Only common tests should be described solely by name; describe more complex techniques in the Methods section.*
☒	☐	A description of all covariates tested
☐	☒	A description of any assumptions or corrections, such as tests of normality and adjustment for multiple comparisons
☐	☒	A full description of the statistical parameters including central tendency (e.g. means) or other basic estimates (e.g. regression coefficient) AND variation (e.g. standard deviation) or associated estimates of uncertainty (e.g. confidence intervals)
☒	☐	For null hypothesis testing, the test statistic (e.g. F, t, r) with confidence intervals, effect sizes, degrees of freedom and P value noted<br>*Give P values as exact values whenever suitable.*
☒	☐	For Bayesian analysis, information on the choice of priors and Markov chain Monte Carlo settings
☒	☐	For hierarchical and complex designs, identification of the appropriate level for tests and full reporting of outcomes
☒	☐	Estimates of effect sizes (e.g. Cohen's d, Pearson's r), indicating how they were calculated

Our web collection on statistics for biologists contains articles on many of the points above.

Software and code

Policy information about availability of computer code

Data collection	No software used for data collection.
Data analysis	All brightfield images were analyzed with "ImageJ 1.53c" software. Print head trajectories was generated using "AutoCAD 2014" software.

For manuscripts utilizing custom algorithms or software that are central to the research but not yet described in published literature, software must be made available to editors and reviewers. We strongly encourage code deposition in a community repository (e.g. GitHub). See the Nature Portfolio guidelines for submitting code & software for further information.

Data

Policy information about availability of data

All manuscripts must include a data availability statement. This statement should provide the following information, where applicable:

- Accession codes, unique identifiers, or web links for publicly available datasets
- A description of any restrictions on data availability
- For clinical datasets or third party data, please ensure that the statement adheres to our policy

The data that support the plots within this paper and other finding of this study are presented in the main article and the Supplementary Materials. All other additional data are available from the corresponding author upon request. The Source Data for this paper is provided in the form of an .xlsx file.

Research involving human participants, their data, or biological material

Policy information about studies with human participants or human data. See also policy information about sex, gender (identity/presentation), and sexual orientation and race, ethnicity and racism.

Reporting on sex and gender	N/A
Reporting on race, ethnicity, or other socially relevant groupings	N/A
Population characteristics	N/A
Recruitment	N/A
Ethics oversight	N/A

Note that full information on the approval of the study protocol must also be provided in the manuscript.

Field-specific reporting

Please select the one below that is the best fit for your research. If you are not sure, read the appropriate sections before making your selection.

☒ Life sciences ☐ Behavioural & social sciences ☐ Ecological, evolutionary & environmental sciences

For a reference copy of the document with all sections, see nature.com/documents/nr-reporting-summary-flat.pdf

Life sciences study design

All studies must disclose on these points even when the disclosure is negative.

Sample size	The samples used for testing in this research were prepared according to the procedure outlined in the Methods section. Each sample was replicated a minimum of three times, guaranteeing sufficient data for accurate statistical analysis.
Data exclusions	No data was ignored in this study.
Replication	All experiments were conducted using independent replicates, as shown in the legend.
Randomization	All samples were randomly assigned to the experimental group.
Blinding	All analyses were blinded with respect to the experimental conditions.

Reporting for specific materials, systems and methods

We require information from authors about some types of materials, experimental systems and methods used in many studies. Here, indicate whether each material, system or method listed is relevant to your study. If you are not sure if a list item applies to your research, read the appropriate section before selecting a response.

Materials & experimental systems

n/a	Involved in the study	
☐	☒	Antibodies
☐	☒	Eukaryotic cell lines
☒	☐	Palaeontology and archaeology
☐	☒	Animals and other organisms
☒	☐	Clinical data
☒	☐	Dual use research of concern
☒	☐	Plants

Methods

n/a	Involved in the study	
☒	☐	ChIP-seq
☒	☐	Flow cytometry
☒	☐	MRI-based neuroimaging

Antibodies

Antibodies used	rabbit anti-F4/80 antibody (70076S, CST)<br>rabbit anti-Ly6G antibody (87048S, CST)<br>rabbit anti-CD206 antibody (24595T, CST)

Validation	Validation of each antibody was done under standard information offered by the supplier.

Eukaryotic cell lines

Policy information about cell lines and Sex and Gender in Research

Cell line source(s)	HEK 293T cell lines were purchased from ATCC company.
Authentication	HEK 293T cell lines were not authenticated by the investigators. They were purchased from commercial company.
Mycoplasma contamination	All cell lines were negative for mycoplasma contamination.
Commonly misidentified lines (See ICLAC register)	No commonly misidentified lines were used in the study.

Animals and other research organisms

Policy information about studies involving animals; ARRIVE guidelines recommended for reporting animal research, and Sex and Gender in Research

Laboratory animals	Female C57 black 6 mice (8 weeks old) were used for all experiments in this study. The mice were housed in a controlled environment with a 12h-12h light-dark cycle (light cycle: 8:00 to 20:00), and they had access to food and water ad libitum. The room temperature was maintained within the range of 20-22°C, while the humidity was kept between 30-70%, with an average daily humidity level of approximately 47%.
Wild animals	No wild animals were used in this study.
Reporting on sex	All female animals were used in this study, and gender differences were not considered.
Field-collected samples	This study did not involve samples collected from field.
Ethics oversight	All studies of implantation experiments in vivo were approved with the permission of the Sichuan University Ethics Committee (protocol number 2020361A) and were conducted according to the institution's guidelines.

Note that full information on the approval of the study protocol must also be provided in the manuscript.
